# Unconventional Yeasts Are Tolerant to Common Antifungals, and *Aureobasidium pullulans* Has Low Baseline Sensitivity to Captan, Cyprodinil, and Difenoconazole

**DOI:** 10.3390/antibiotics9090602

**Published:** 2020-09-15

**Authors:** Electine Magoye, Maja Hilber-Bodmer, Melanie Pfister, Florian M. Freimoser

**Affiliations:** 1 Agroscope, Research Division Plant Protection, Müller-Thurgau-Strasse 29, 8820 Wädenswil, Switzerland; electine.magoye@agroscope.admin.ch (E.M.); maja.hilber-bodmer@agroscope.admin.ch (M.H.-B.); 2 Swiss Federal Institute of Technology (ETH) Zurich, 8092 Zurich, Switzerland; melanie.pfister@biol.ethz.ch

**Keywords:** fungicide, resistance, baseline sensitivity, yeasts, isolation, captan, cyprodinil, difenoconazole

## Abstract

Many yeasts have demonstrated intrinsic insensitivity to certain antifungal agents. Unlike the fungicide resistance of medically relevant yeasts, which is highly undesirable, intrinsic insensitivity to fungicides in antagonistic yeasts intended for use as biocontrol agents may be of great value. Understanding how frequently tolerance exists in naturally occurring yeasts and their underlying molecular mechanisms is important for exploring the potential of biocontrol yeasts and fungicide combinations for plant protection. Here, yeasts were isolated from various environmental samples in the presence of different fungicides (or without fungicide as a control) and identified by sequencing the internal transcribed spacer (ITS) region or through matrix-assisted laser desorption/ionization time-of-flight (MALDI-TOF) mass spectrometry. Among 376 isolates, 47 taxa were identified, and *Aureobasidium pullulans* was the most frequently isolated yeast. The baseline sensitivity of this yeast was established for 30 isolates from different environmental samples in vitro to captan, cyprodinil, and difenoconazole. For these isolates, the baseline minimum inhibitory concentration (MIC_50_) values for all the fungicides were higher than the concentrations used for the control of plant pathogenic fungi. For some isolates, there was no growth inhibition at concentrations as high as 300 µg/mL for captan and 128 µg/mL for cyprodinil. This information provides insight into the presence of resistance among naturally occurring yeasts and allows the choice of strains for further mechanistic analyses and the assessment of *A. pullulans* for novel applications in combination with chemical agents and as part of integrated plant-protection strategies.

## 1. Introduction

Fungicide resistance is an extremely important issue in medicine as well as agriculture. In both settings, the application of fungicides favours the selection of resistant strains that can consequentially become serious threats for human or crop health. Owing to these threats, fungicide resistance in human and plant pathogenic fungi is well studied at all levels, from their ecological impact and population dynamics to the molecular mechanisms involved [1,2,3,4,5,6,7]. By contrast, fungicide resistance and sensitivity in non-target fungal species is much less investigated. This is somewhat surprising, because these non-target fungi may reveal inherent resistance mechanisms, provide sources of resistance genes, or lead to new applications where fungicide tolerance may be a desirable trait (e.g., the decomposition of fungicides or combinations of fungicides and tolerant isolates for biocontrol applications). For example, the combinations of wild yeasts (*Rhodotorula mucilaginosa*, *R. glutinis*, and *R. graminis*) with several chemical fungicides was more effective in controlling *Botrytis cinerea* than either fungicide or biocontrol yeast alone [8]. Similar combined applications to manage head blight, powdery mildew, or different fruit-decay diseases have subsequently been reported [9,10,11,12,13,14]. It may thus be possible to develop new, commercial plant-protection strategies employing combinations of biocontrol yeasts and fungicides in order to reduce the total application rate of the latter and the development of fungicide-resistant plant pathogens.

Most fungicides used for crop protection are threatened by the development of resistance of the respective fungal pathogens [15,16,17]. The levels of risk vary among different fungicide chemical groups [18]. Risk management is imperative, especially in fungicide groups with a high risk of resistance development, but also needed for fungicides in medium- or low-risk groups to avoid the introduction or progression of resistance. For the study described here, we choose the three fungicides captan (CPN, phthalimide class of fungicides), cyprodinil (CYP, anilinopyrimidine class of fungicides), and difenoconazole (DFN, demethylation inhibitor (DMI) fungicide class) as representatives for commonly used fungicide classes. With respect to the risk of resistance development, these three fungicides belong to either medium- or low-risk groups [19]. CPN has multiple targets in the cell, but its exact mode of action is poorly described [18]. The multiple targets of CPN are likely the cause of the low risk of resistance development and the limited number of resistant strains that have been documented [19]. Still, there are some reports of reduced sensitivity to captan [20,21]. CPN is employed to control scab, blights, and shot hole diseases in apples, pears, cherries, and stone fruits. CYP is used to control scab and rot diseases of stone and pome fruits. Examples include apple (*V. inaequalis*) and pear scab (*V. pirina*), brown rot (*Monilinia fructicola*), and blossom blight (*Monilinia laxa*) in plums, apricots, peaches, and nectarines; and diseases caused by *B. cinerea* (e.g., *Botrytis* bunch rot and *Botrytis* fruit rot in pome fruits). Anilinopyrimidines are considered to have a medium risk for the development of resistance [18]. To date, resistance development has, for example, been reported in *Venturia* spp. and *Botrytis* spp. DFN is registered for controlling diseases such as carrot black leaf and pod spot (*Alternaria* spp.), powdery mildew (*Podosphaera* spp. and *Erysiphe* spp.), scabs (*V. inaequalis* and *V. pirina*), and rots and blights *(Monilinia* spp.) in different crops. DMI fungicides are a widely used class of fungicides and, despite their widespread use, still considered to have a medium risk of resistance development [18].

Few species of unconventional, non-pathogenic yeasts are currently being used in agriculture and biotechnology, but new activities and potential applications are described for a plethora of such yeasts. Many species are used for food and beverage production or as sources of enzymes and valuable chemicals. For example, the yeast *Aureobasidium pullulans* has antifungal or antibacterial properties, is commercially used as a biocontrol agent, and produces a range of metabolites that are of biotechnological interest [22,23,24,25]. Besides *A. pullulans*, *Candida oleophila*, *Metschnikowia fructicola*, *Saccharomyces cerevisiae*, and *Cryptococcus albidus* are or have been registered in different biocontrol products [25]. In addition, some yeasts and yeast products have been explored in novel applications (e.g., in combination with antifungal/antibacterial formulations) to manage plant diseases [8,9,10,11,12,13,14,24,26]. However, to fully exploit the potential of unconventional yeasts for such combined applications, it is key to understand how frequent fungicide-insensitive yeasts are and what the functional or molecular mechanisms underlying this phenotype are. Although CPN, CYP, and DFN have been registered and utilised in the environment, and the mechanisms of resistance have been studied in plant pathogenic fungi, little is known about the tolerance to these fungicides for unconventional, non-pathogenic yeasts. To understand the distribution of fungicide sensitivity in natural populations and the development of fungicide resistance, it is important to establish the baseline sensitivities of natural isolates to different fungicides [27]. For many pathogenic fungi, the baseline sensitivities for CPN, CYP, and DFN have already been determined [20,28,29,30,31], but this is not the case for non-pathogenic, naturally occurring yeasts.

The objectives of this study were, therefore, to isolate and identify naturally occurring yeasts that are tolerant to commonly used antifungals (agricultural and medical) and to establish the baseline sensitivities for CPN, CYP, and DFN for the most frequently isolated yeast, *A. pullulans*.

## 2. Results

### 2.1. Isolation and Identification of Naturally Occurring Yeasts Insensitive to Commonly Used Fungicides

#### 2.1.1. Yeasts Are Tolerant to Commonly Used Antifungal Agents

**Figure 1:**
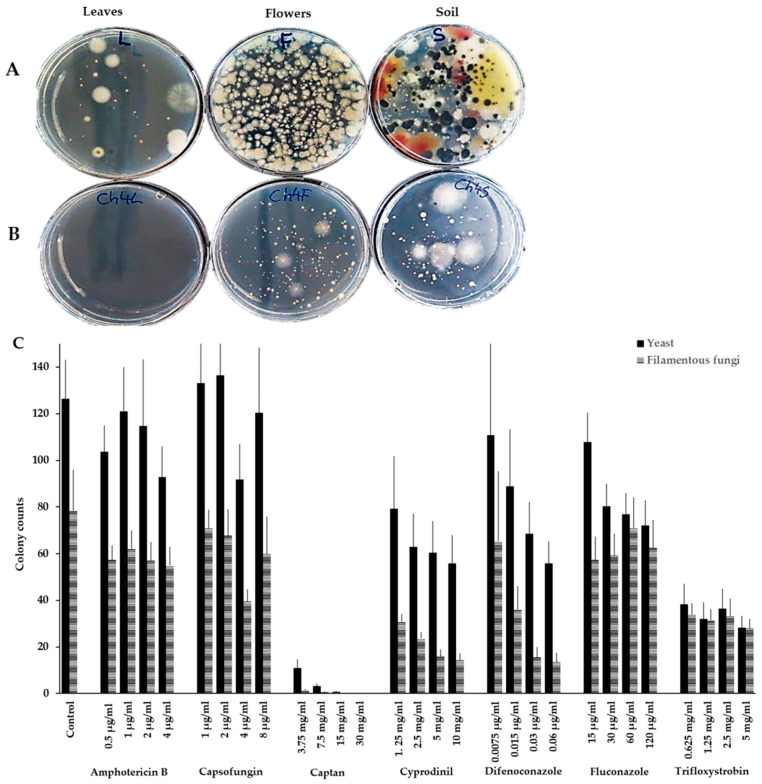
Yeasts tolerate commonly used antifungal agents. Yeasts from soil were isolated in 1% peptone water. These suspensions were then incubated for 1 h in 1% peptone water (**A**) or in peptone water containing different antifungal agents (e.g., 10 mg/mL cyprodinil (CYP)) (**B**). The mixtures were plated on antibiotic-supplemented potato dextrose agar (PDA) plates and incubated for 72 h. Fewer fungal colonies were isolated in the presence of antifungals as compared to the control. Colonies were counted on the control and fungicide plates. An example portraying the colony counts for filamentous fungi and yeasts in a soil sample is shown (**C**). The number of yeast colonies was consistently higher than that of filamentous fungi. The chart shows the average numbers and standard errors for the corresponding colony counts for three separate soil isolations, with three replicates each. All the data were pooled.

Different antifungal agents, in different concentrations, were mixed with environmental samples (soil, leaves, flowers, and fruits), the suspensions were plated on potato dextrose agar (PDA) (containing antibiotics to prevent the growth of bacteria), and fungal colonies were counted and isolated. Many fungal colonies (both filamentous fungi and yeasts) were observed on the control plates, on which the samples without any antifungal agent were plated (Figure 1A). The total number of fungal colonies was reduced in the samples isolated in the presence of the antifungals amphotericin B, capsofungin, CPN, CYP, DFN, fluconazole, and tryfloxystrobin (Figure 1B). Interestingly, in the presence of all these fungicides except trifloxystrobin, the number of yeast colonies was consistently higher as compared to the colony counts for filamentous fungi (Figure 1C). For CPN, CYP, DFN, and fluconazole (for yeasts only), the total number of fungal colonies declined as the antifungal concentrations increased, but for the other compounds, this effect was not clearly observed (Figure 1C). Notably, medically used antifungals (i.e., amphotericin B, capsofungin, and fluconazole) had the least effect on overall fungal colonies, while captan had the most potent effect, resulting in almost no filamentous fungi and very few yeast colonies (Figure 1C). Overall, these results indicate that environmental yeasts can tolerate the presence of most of the fungicides tested here.

#### 2.1.2. *Aureobasidium pullulans* Is the Most Frequently Isolated Species in the Presence of Antifungals

**Figure 2:**
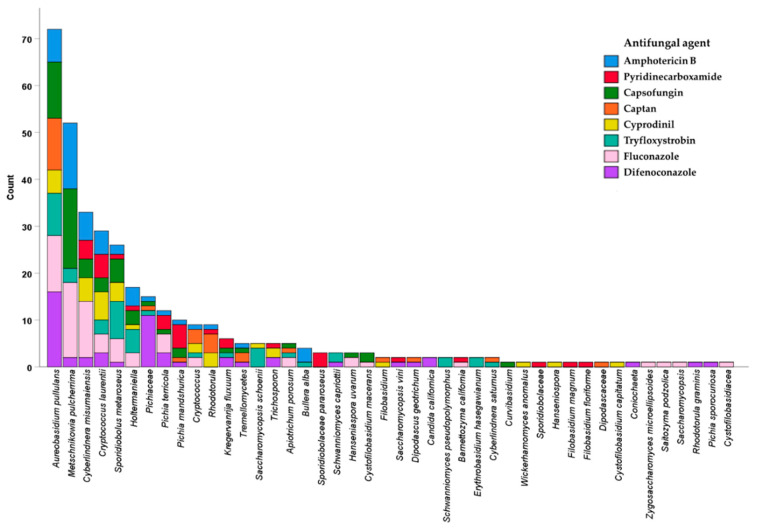
*Aureobasidium pullulans* is most frequently isolated species in the presence of fungicides. All the yeast taxa (species hypotheses, here referred to as species) that were identified are listed on the *X*-axis, while the *Y*-axis indicates the number of isolates obtained for each species. The color codes represent the fungicides used during the isolation, with the corresponding total numbers of isolates.

Single yeast colonies were picked from plates with the highest antifungal concentrations where yeasts were still present and used for identification. A total of 359 yeast isolates belonging to 48 taxa were identified after isolation in the presence of different antifungal agents (Figure 2)*. A. pullulans* was the most abundantly isolated yeast with seven out of the eight antifungal agents (73 isolates in total), while some species were isolated only once and only with a single antifungal (Figure 2). Other commonly isolated taxa (at least 16 or more isolates, in the presence of at least five different fungicides) included *M. pulcherrima, Cryptococcus laurentii, Cyberlindnera misumaiensis, Sporidiobolus metaroseus*, and *Holtermaniella. Pichiaceae* were mostly isolated with DFN (eleven out of the total fifteen *Pichiaceae* isolates). The different yeast species were naturally occurring both in the phyllosphere (leaves, flowers, and fruits) and in the soil, but a broader diversity (38 species) was observed in the soil samples (Table 1). Interestingly, *A. pullulans, S. metaroseus,* and *Holtermaniella* were found in all the four sample types, while *M. pulcherrima* was found in all the samples except in flowers. The numbers of isolates ranged from 11 to 66 for each antifungal agent used (Supplementary Table S1). Seasonal changes affected the total number but not species diversity, since most species were collected throughout the four seasons.

### 2.2. The Baseline Sensitivities and MIC_50_ of 30 A. pullulans Isolates

**Figure 3:**
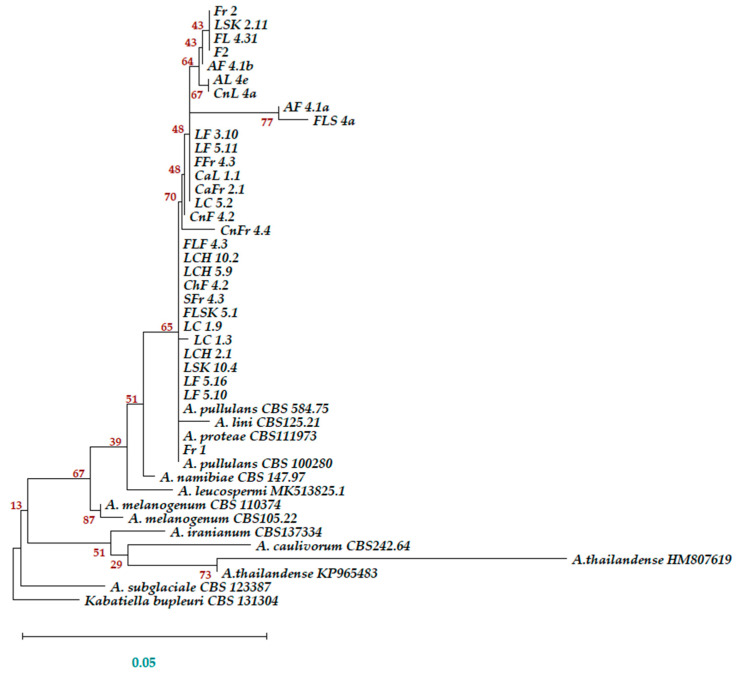
The ITS sequences of the 30 *A. pullulans* isolates cluster with published *A. pullulans*, *A. proteae*, and *A. lini* ITS sequences. The evolutionary history was inferred by using the Maximum Likelihood method and Tamura–Nei model [35]. The tree with the highest log likelihood (−1641.85) is shown. The percentages of trees in which the associated taxa clustered together are shown next to the branches. The initial tree for the heuristic search was obtained automatically by applying the Maximum Parsimony method. A discrete Gamma distribution was used to model evolutionary-rate differences among sites (5 categories (+G, parameter = 0.2691)). The rate variation model allowed for some sites to be evolutionarily invariable ([+I], 0.00% sites). The tree is drawn to scale, with branch lengths representing the numbers of substitutions per site. This analysis involved 44 nucleotide sequences. There were a total of 675 positions in the final dataset. Evolutionary analyses were conducted in MEGA X [36].

Since *A. pullulans* was the most abundantly isolated yeast, a large number of isolates allowing the determination of baseline sensitivities to different fungicides was available. Thirty isolates of this species were thus selected for further analysis. To assess their diversity and relationship with known *Aureobasidium* strains, the ITS sequences of these 30 isolates were used for a phylogenetic analysis. Based on their ITS sequences, 30 of these isolates clustered together with other, already published, *Aureobasidium* strains (Figure 3). This cluster comprised *A. pullulans*, but also species such as *A. proteae* and *A. lini*. However, the 30 isolates did not cluster with *A. namibiae*, *A. melanogenum*, and *A. sublgaciale*, which were defined as separate species [34]. Since the 30 isolates were identified as *A. pullulans* by the UNITE database (as SH1515060.08FU) and all the ITS sequences formed a cluster that also included the sequence of the neotype for *A. pullulans* var. *pullulans*, CBS 584.75, these isolates were treated as *A. pullulans* for this study.

In order to assess the fungicide sensitivity of the 30 *A. pullulans* isolates, extensive microbroth sensitivity growth assays in the presence of different concentrations of three fungicides were performed. The 30 isolates of *A. pullulans*, isolated under different conditions (sample sources, time points, and fungicides) were controlled (i.e., at least 50% growth reduction) by the experimental concentrations of CPN and DFN and the majority of the isolates (63% and 70%, respectively) had a MIC_50_ value that was below the mean MIC_50_ for the corresponding fungicide (Table 2). Additionally, for CYP, the majority of the isolates (70%) had a MIC_50_ below the mean for CYP. However, not all the isolates were controlled: isolate AL4e was insensitive to the maximal CYP concentration used here (256 µg/mL) and had a calculated MIC_50_ value of 1.45 × 10^39^ µg/mL. This value was thus excluded from further calculations. The resistance factors (i.e., the maximum MIC_50_ value divided by the minimum MIC_50_ value) were lower for difenoconazole (25.3) and captan (21.5) than for cyprodinil (93.0). Overall, the 30 isolates had mean MIC_50_ of 28.9 (CPN), 22.6 (CYP), and 2.2 µg/mL (DFN) (median MIC_50_ were 21.9, 8.9, and 1.4 µg/mL for CPN, CYP, and DFN, respectively). DFN had the narrowest MIC_50_ range (0.4–10.1 µg/mL), followed by CPN (5.1–109.6 µg/mL), while CYP had the widest range (2.0–186.0 µg/mL).

**Figure 4:**
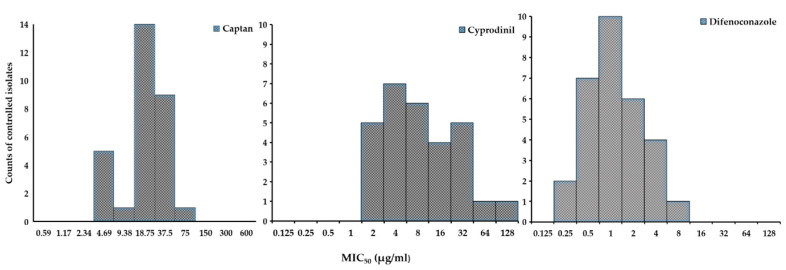
The frequencies of the MIC_50_ values for the 30 *A. pullulans* isolates show non-normal distributions. The sensitivities of the 30 *A. pullulans* isolates to CPN, CYP, and DFN were determined in microbroth sensitivity assays. The minimum concentration inhibiting the growth of the yeasts by at least 50% (as determined by OD600 measurements; MIC_50_) was calculated. The numbers of isolates with a particular MIC_50_ value are plotted.

The distributions of the MIC_50_ values for all the three fungicides and the 30 isolates were skewed, since many isolates exhibited an increased sensitivity (i.e., had a lower MIC_50_ value) compared to the average for the studied population (Figure 4, Table 2). The non-transformed MIC_50_ values for all the three fungicides thus resulted in non-normal distributions (Shapiro–Wilk W = 0.82, *p* = 0.0001 (CPN); W = 0.51, *p* < 0.001 (CYP); W = 0.75, *p* < 0.001 (DFN)). Overall, the distributions of the MIC_50_ values were unimodal, potentially indicating that no disruptive resistance existed and that the *Aureobasidium* population studied here showed baseline sensitivity with significant variation.

The mean MIC_50_ values for the control of the *A. pullulans* isolates were compared with the EC_50_ values reported for applications against plant pathogenic fungi. For the control of *B. cinerea*, mean EC_50_ values of 0.9 (CPN) and 0.008 µg/mL (CYP) have been reported, while for DFN, the mean EC_50_ for controlling *Penicillium* spp. was 0.16 µg/mL [28,37,38]. The mean MIC_50_ values for the *A. pullulans* isolates were thus significantly higher (CPN: T = 7.33, df = 29, *p* < 0.001; DFN: T = 5.27, df = 29, *p* < 0.001; CYP: T = 3.41, df = 28, *p* = 0.002) than the published mean EC_50_ values for plant pathogenic fungi, suggesting that *A. pullulans* is less sensitive to these three fungicides than the plant-pathogen targets of the respective fungicides.

### 2.3. The MIC_50_ Values for the 30 A. pullulans Isolates for CPN, CYP, and DFN Show a Significant, Positive Correlation

The 30 *A. pullulans* strains were initially isolated in the presence of different fungicides. However, a relationship between the initial fungicide used for isolation and the MIC_50_ values for CPN, CYP, and DFN was not apparent. For example, for CPN, the most tolerant isolate was initially isolated in fluconazole. Interestingly, three of the five strains isolated in the presence of CPN (i.e., LC 1.3, LC 1.9, and LC 5.2, all isolated from leaves) exhibited low sensitivity to all three fungicides. Isolates LCH 10.2, LCH 5.9, LCH 2.1, and ChF 4.2 were isolated with CYP but were not among the isolates most tolerant to this fungicide. By contrast, the most CYP-tolerant isolate AL 4e had initially been isolated in amphotericin B. Finally, an isolate initially isolated in DFN (e.g., SFr 4.3, LSK 2.11, FLSK 5.1, and LSK 10.4) was not more tolerant to DFN than other isolates (Table 2), likely suggesting that a pleiotropic mechanism of tolerance towards fungicides in general, as opposed to a specific resistance mechanism against a particular agent, is involved.

Thus far, we have determined the MIC_50_ values for 30 *A. pullulans* strains for three fungicides and identified low sensitivity to CPN, CYP, and DFN in at least some isolates. In order to assess if tolerance was fungicide specific or if the same isolates were either sensitive or tolerant to all fungicides, the Pearson correlation coefficients (r) among the MIC_50_ values for all the isolates and the three fungicides were calculated.

**Figure 5:**
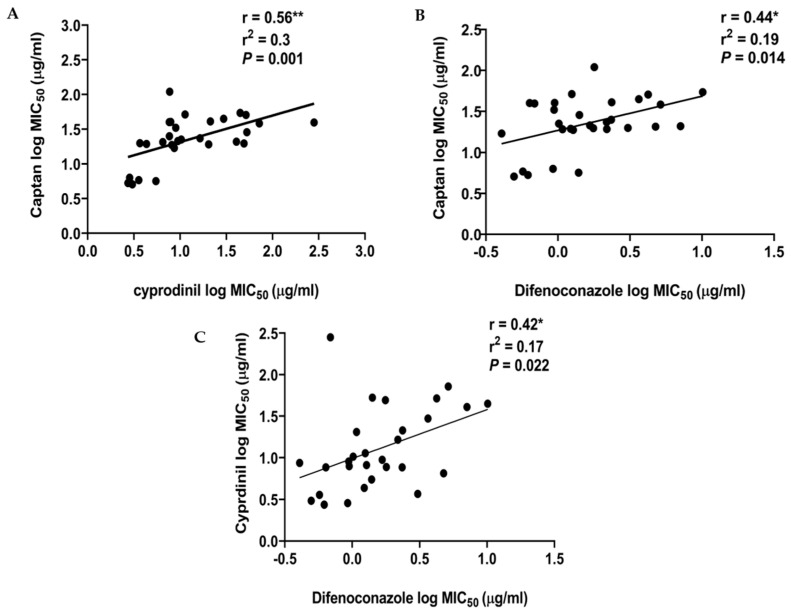
The MIC_50_ values of the 30 *A. pullulans* isolates for CPN, CYP, and DFN show a significant, positive correlation. Pearson’s correlations portraying the relationships between the MIC_50_ values for the *A. pullulans* isolates for (**A**) CPN and CYP, (**B**) CPN and DFN, and (**C**) CYP and DFN. All the relationships were statistically significant. Correlation was determined using the log-transformed MIC_50_ values, assuming a Gaussian distribution of the data, in which the outlier (isolate AL4e with CYP) was removed. The interpolation line was fitted with linear regression (r^2^) [39].

In all three comparisons (i.e., CPN–CYP, CPN–DFN, and CYP–DFN,) a weak positive correlation was detected (Figure 5), suggesting that the overall tolerance for these three fungicides correlates. The correlation between the CPN and CYP MIC_50_ values was the strongest (r = 0.56) (Figure 5A), while the CPN and DFN (Figure 5B) and CYP and DFN values (Figure 5C) correlated slightly less (r = 0.43 and 0.38, respectively). All these relationships were statistically significant (*p* < 0.05). Overall, these results implied that tolerance to one fungicide goes along with lower sensitivity to the other two fungicides. Since the three fungicides used here belong to different classes and act on different targets, it is thus likely that the insensitive *A. pullulans* isolates identified here mainly exhibit pleiotropic mechanisms causing multi-drug tolerance.

**Figure 6:**
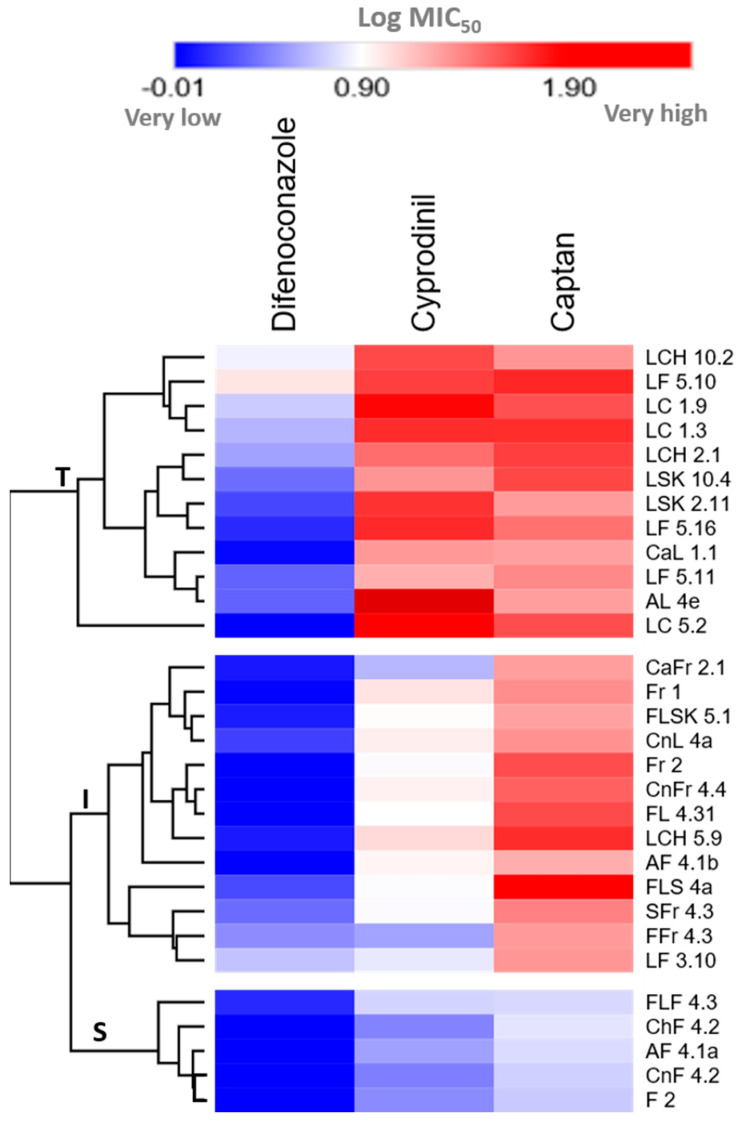
Clustering of the 30 *A.pullulans* isolates into tolerant (T) and sensitive (S; to one or two, or all three fungicides used here) isolates. The dendrogram was plotted using the hierarchical complete linkage clustering method (distance between clusters measured using the Euclidean distance) for the log MIC_50_ values for the three fungicides. The distributions of the sensitivities in the respective clusters based on log MIC_50_ values are highlighted in the heat map. S = sensitive (to all three fungicides), I = intermediate (sensitive to DFN and tolerant to one or two fungicides), and T = tolerant (insensitive to all the three fungicides or to CPN and CYP).

In order to better visualize the different MIC_50_ values and to assess if the *A. pullulans* isolates could be grouped based on their responses to the three fungicides, a heat map was generated and a clustering analysis was performed (Figure 6). This analysis clearly identified a small cluster of highly sensitive isolates (S) that was distinguished from the intermediate and tolerant *A. pullulans* isolates (I and T, respectively). The intermediate cluster (I) had one grouping of isolates sensitive to DFN and CYP, but tolerant to CPN, and a second cluster of isolates sensitive to DFN but tolerant to CPN and CYP. Interestingly, all the isolates in cluster S (sensitive to all the three fungicides) were obtained from flowers, while all the T isolates (tolerant to all the three fungicides or tolerant to CPN and CYP) were sampled from leaves. Overall, these results document differential responses of the 30 *A. pullulans* isolates to the three fungicides and thus suggest that various, general mechanisms are likely to be involved in the insensitivity of many of the isolates studied here. However, to identify the exact mechanisms involved and compare isolates from the different clusters described here, detailed molecular analyses will be required.

## 3. Discussion

Agricultural production requires the management of plant diseases, to both minimise crop losses and maintain crop quality by preventing impacts on humans and the environment as well as the development of fungicide resistance. However, consumers and regulatory agencies demand the minimal use of pesticides and crops, without residues of plant-protection agents. There is thus a strong incentive and pressure to reduce fungicide applications. This can be achieved by either reducing the dosage of fungicides or decreasing the number of applications throughout the season [40,41]. Combining traditional fungicides with a biocontrol agent, such as an antagonistic yeast, in a disease management strategy can either reduce the number of the fungicide applications or allow the reliable application of the minimal effective dosage of the fungicide itself. Such combined treatments have been used not only to lower the number of fungicide applications, but also to reduce resistance selection [41]. Novel yeast–fungicide formulations, thus, may have the potential to reduce the amount of fungicides applied throughout the season. Such applications may also lead to a more reliable efficacy of biocontrol organisms, save time because multiple applications are combined, and reduce chemical residues on crops. Since *A. pullulans* is already a well-established biocontrol agent and some isolates were tolerant to CPN, CYP, or DFN (or even to two or all three of these), this species could be explored for such combined applications. However, before such applications can be put into general practice, the frequency and nature of such insensitivities should be identified in order to be able to properly assess the possible risks (e.g., an increase in and spread of fungicide insensitivity and resistance). Here, we performed the first step of such an assessment by studying naturally occurring yeasts and quantifying fungicide sensitivity in the biocontrol yeast *A. pullulans*.

Wild yeasts were isolated from different agricultural samples in the presence of the fungicides CPN, CYP, and DFN. In total, 376 isolates were obtained, of which 13 different taxa were isolated from apple leaves, while eight and four taxa were obtained from flowers and fruits, respectively (Table 1). By contrast, from soil, 41 different taxa were isolated in the presence of antifungal agents (Figure 1 and Table 1). The larger number of soil yeasts isolated in the presence of fungicides may reflect the higher species diversity in soil as compared to that in the phyllosphere [42]. Soil acts as a reservoir of phyllosphere yeasts and provides a plethora of niches with different nutrients and substrates that soil yeasts can thrive in [43,44,45,46]. It is also possible that some of the many soil fungi bind or inactivate fungicides, thereby reducing their effective concentrations and thus allowing otherwise sensitive species to be isolated. In another study including herbicides, fungicides, and insecticides, only five of 11 yeast species were insensitive to fluquinconazole, while all were sensitive to prochloraz [47]. Among phyllosphere yeasts, only the four species *M. pulcherrima*, *A. pullulans*, *Pichia anomala*, and *S. cerevisiae* were identified as resistant to pesticides [48]. However, sensitivity profiles for medical antifungals have been determined for several *A. pullulans* and *Cryptococcus* isolates. Similar to that in the study presented here, the MIC_50_ for fluconazole for these environmental yeasts was higher compared to that for medically relevant yeasts [49,50].

*A. pullulans* was, by far, the most frequent species (76 isolates), isolated in the presence of seven out of the eight antifungal agents tested and found in all the four sample sources (soil, leaves, fruits, and flowers). This highlights the ubiquitous nature of *A. pullulans* and its ability to thrive in different habitats (e.g., soil, leaves, flowers, and fruits) and environmental conditions (e.g., hypersaline habitats, glaciers, arid conditions, and radiation sites) due to the presence of genes that confer stress tolerance [34,46,51,52,53,54,55]. Similarly, *M. pulcherrima, C. laurentii, C. misumaiensis,* and *S. metaroseus* are also commonly occurring and frequently isolated from the leaves of various trees, fruits, and soils of both agricultural and wild habitats, and can tolerate extreme conditions [43,46,51,56,57,58,59]. Their frequent isolation likely represents the high abundance of these species in the environment but also, likely, their tolerance to the antifungals used for isolation. Interestingly, though, the CPN, CYP, and DFN sensitivities of the 30 *A. pullulans* isolates studied here were not reflected in the initial fungicide used for isolation. For example, the isolate most tolerant to CYP was not isolated in the presence of CYP, but was in that of fluconazole. This likely implies that these yeasts rather exhibit a pleiotropic mechanism of tolerance towards fungicides as opposed to a specific resistance mechanism against a particular agent. It thus seems that high abundance, stress tolerance, and competitiveness in a broad range of environments go hand in hand with low sensitivity to antifungal agents. The unique biochemical and genetic properties rendering these yeasts particularly stress tolerant may thus also confer a general, unspecific insensitivity to antifungal compounds [60].

The mean baseline MIC_50_ values that were determined here for the 30 *A. pullulans* isolates and the three different fungicides CPN, CYP, and DFN were higher than the concentrations of the corresponding fungicides used in the field to control plant pathogens. This was particular striking for CYP, where the mean baseline MIC_50_ was 22.6 µg/mL and thus significantly higher than the concentration of 0.008 µg/mL that is used in the field against the plant pathogen *B. cinerea* [37,38] and the low EC_50_ values of some plant pathogens [61]. Similarly, the mean baseline sensitivity for DFN was 2.18 µg/mL and statistically higher than the mean EC_50_ for the control of 97 *Penicilium* spp. (0.16 µg/mL) or 44 *V. inaequalis* isolates (0.002 µg/mL) [28,31]. Although this baseline for DFN was higher for *A. pullulans* isolates, all of the isolates were controlled by DFN and only nine isolates out of the 30 showed reduced sensitivity (had MIC_50_ values above the mean). For CPN, the mean MIC_50_ value for the 30 *A. pullulans* isolates tested here was 28.9 µg/mL and thus also significantly higher than the mean EC_50_ of wildtype and resistant *B. cinerea* (0.9 and 5 µg/mL, respectively) [20,37,38]. Overall, the *A. pullulans* MIC_50_ values for CPN and DFN, CPN and CYP, and CYP and DFN correlated positively (weakly, but statistically significantly), which may also indicate a general mechanism of insensitivity of *A. pullulans* to these fungicides.

None of the three fungicides CPN, CYP, or DFN harbours a particularly high risk for the development of resistance by plant pathogenic fungi. CPN is highly effective in controlling plant pathogenic fungi, and the risk of resistance development seems low [18,19,62,63]. Nevertheless, resistance to CPN was reported after the in vitro testing of *B. cinerea* isolates from different orchards in Canada and from commercial blueberry fields in Florida [20,21]. One resistance mechanism for CPN is the increased biosynthesis of molecules containing thiol groups (i.e., glutathione), which has been described for *B. cinerea* [21] but could also be a mechanism rendering *A. pullulans* less sensitive to this fungicide. The trichloromethylthiol group of CPN non-enzymatically and irreversibly reacts with exposed thiol groups, resulting in a thiophosgene moiety and tetrahydrophthalimide (THPI) [64,65]. CPN is also sensitive to and unstable at high pH [66]. Therefore, the insensitivity of *A. pullulans* isolates to CPN might be due to the increased production of molecules with exposed thiol groups, a loss of stability in culture supernatants (e.g., due to an increase in pH), or the degradation of CPN. More detailed studies are, however, needed to understand if one or more of these mechanisms are the cause of the insensitivity of *A. pullulans* to CPN and also to identify the mechanisms conferring insensitivity to all three fungicides tested here. Resistance to CYP is rare in most orchards in the US and Europe, with the sensitivity thresholds for different pathogenic fungi in both regions set to between 0.03 and 5 mg/L [20,29,30,67,68]. Nevertheless, resistance has been noted and attributed to point mutations in the *BcmetB* gene and in nine different genes that encode mitochondrial proteins [69,70,71]. The wide range of MIC_50_ values for CYP that were determined for *A. pullulans* (2.82–186 µg/mL) may be explained by the complex mode of action of CYP. Resistance against DFN has been reported in laboratory-induced mutants. The mutation of tyrosine to phenylalanine at codon 126 (Y126F) in the Cyp51 protein of *Pencillium expansum* and increased expression levels of the *CYP51A1* gene were identified to correlate with DFN resistance [72,73]. Field resistance to DFN is still low but predicted to increase if proper resistance-management practices are not reinforced [74]. To extend DFN’s life span, it is applied as a mixture with other compounds. The fact that the yeasts known to be particularly stress resistant seemed to be particularly insensitive to the fungicides and the positive correlation between the insensitivities to CPN, CYP, and DFN seem to suggest that *A. pullulans* is, in general, fungicide tolerant. Detailed studies at the molecular level will identify if this is indeed the case or if insensitivity correlates with specific mutations.

In summary, this study documents the widespread insensitivity of naturally occurring yeasts to different antifungals and highlights the remarkable fungicide insensitivity of at least some *A. pullulans* isolates. This property is a precondition for possible combinations and the synergistic action of a biocontrol agent and a fungicide. Since several *A. pullulans* isolates were tolerant to even the highest concentration of CYP used in the field, a combined disease-management approach (*A. pullulans* as a biocontrol agent and CYP) could be envisioned for plant protection. In general, such biocontrol–fungicide combinations may not only allow reducing the amount of fungicides applied in the field but also prevent the development of resistance against fungicides. To slow down the development of fungicide resistance and prolong the effective lifetime of a fungicide, the use of antifungal agents with different modes of action (either simultaneously, sequentially, or in a single formulation) is recommended [75,76,77]. For example, DFN and CYP have been combined in a single formulation, marketed as InspireSuper^®^ (Syngenta), and used to efficiently manage disease [29]. Since we identified several *A. pullulans* isolates that exhibited low sensitivity to CPN, CYP, and DFN, we may even envision a combination of *A. pullulans* with two different fungicides. However, more studies are still necessary to understand the particular mechanisms that render *A. pullulans* tolerant to CPN, CYP, and DFN and to assess the potential applications of biocontrol–fungicide combinations in plant protection.

## 4. Materials and Methods

### 4.1. Fungal Isolate Collection and Storage

Environmental samples (cherry fruits; apple leaves and flowers; and soil from different apple and cherry orchards in Wädenswil, Switzerland) were collected from October 2018 to July 2019. Sampling was mainly performed in orchards that had never been treated with fungicides, but some samples were obtained from fields that had been treated. Amounts of 1 g of soil samples or 2 g of leaves, flowers, or fruits were mixed with 10 mL of 1% peptone water (Carl Roth GmBH, Karlsruhe, Germany) and incubated for 30 min (with vigorous shaking on an orbital shaker (Ecotron^®^, Infors-ht, Bottmingen, Switzerland) at 200 rpm and 22 °C). Yeasts were isolated in the presence of different, commercially available fungicides (amphotericin B, capsofungin, fluconazole, and 8-hydroxyquinoline sulfate (Fisher Scientific AG, Basel, Switzerland); Chorus^®^ (50% cyprodinil), Slick^®^ (250 g/L difenoconazole), and Captan 80 WDG (80% captan) (Syngenta AG, Basel, Switzerland); Flint^®^ 500 WG (500 g/kg trifloxystrobin) (Bayer Crop Science); and boscalid (pyridine carboxamide) (BASF). The final concentrations for the seven fungicides were as follows: amphotericin B (4, 2, 1, and 0.5 µg/mL); fluconazole (120, 60, 30, and 15 µg/mL); capsofungin (8, 4, 2, and 1 µg/mL); Slick (0.06, 0.03, 0.015, and 0.0075 µg/mL); chorus (10, 5, 2.5, and 1.25 mg/mL); flint (5,2.5, 1.25, and 0.625 mg/mL); captan (30, 15, 7.5, and 3.75 mg/mL); boscalid (10, 20, 40, and 80 µg/L); 8-hydroxyquinoline sulfate (7.5, 15, 30, and 75 mg/mL). The samples were incubated for 1 h (with shaking on an orbital shaker at 22 °C and 200 rpm). An aliquot of 50 µL (25 µL for soil samples) of each dilution was plated on potato dextrose agar (PDA; Difco) dishes supplemented with chloramphenicol (0.5%) and tetracycline (0.5%) and incubated at 22 °C for 72 h. This procedure was performed in five replicates and in a manner that yielded single, well-separated fungal colonies. After incubation, yeast and filamentous colonies were counted. Yeast colonies from each replicate plate were selected (based on different morphological characteristic) and purified by sub-culturing twice on PDA to obtain pure cultures. All the isolates were stored at −80 °C in 15% (*v*/*v*) glycerol.

### 4.2. Fungal Identification

As a faster and more economical alternative to DNA sequencing, yeast identification was first attempted using matrix-assisted laser desorption/ionization time-of-flight mass spectrophotometry (MALDI-TOF MS) as previously described [46,78], with a few modifications. Single yeast colonies were transferred onto an AXIMA-CFR MALDI-TOF target plate (Kratos, Manchester, UK) using a toothpick. The smears were left to air dry and then overlaid with 1 μL of matrix (Sinapinic acid (SA), 40 µg/mL in acetonitrile–ultra pure water (UPW)–trifluoroacetic acid (TFA) (0.6:0.4:0.003) per mL). The SA, acetonitrile, and TFA were purchased from Sigma-Aldrich Chemie GmbH, Steinheim, Germany; the UPW was produced by an Arium^®^ water filter system. To create the MALDI–TOF MS reference spectra, eight replicates of the same species were spotted on the target plate and mass spectra for each spot were obtained using an AXIMA Performance MALDI-TOF MS machine (Shimadzu Schweiz GmbH, Reinach, Switzerland). All the spectra were analysed using the inbuilt AXIMA microorganism identification system (Shimadzu Schweiz GmbH). For species that could not be identified by MALDI-TOF MS, the ITS region was amplified and sequenced as previously described [79]. All the *A. pullulans* isolates studied in detail in this work were identified by sequencing the ITS region. The sequences were processed and analysed using the Genious™ software, and all the sequenced isolates were assigned a species hypothesis (SH) number according to the UNITE database [80] (see also Supplementary Table S1).

### 4.3. Determination of Baseline MIC_50_ Values

Thirty isolates of *A. pullulans,* isolated in the presence of different fungicides and from a variety of sources and locations (Table 3), were tested for sensitivity to CPN, CYP, and DFN using the microbroth sensitivity assay.

Concentrated stock solutions (5.12 mg/mL) of technical-grade DFN and CYP (Sigma-Aldrich Chemie, Schweiz, Buchs, Switzerland) were prepared in acetonitrile and methanol, respectively, and serially diluted (1:2) with the respective solvents to achieve 2× the final concentrations (0.25, 0.5, 1, 2, 4, 8, 16, 32, 64, 128, and 256 µg/mL). Similarly, technical-grade CPN (Fisher Scientific AG, Reinach, Switzerland) was prepared in acetonitrile, adjusted to a concentrated stock solution (24 mg/mL), and diluted with acetonitrile to achieve 2× the final concentrations of 1.17, 2.34, 4.68, 9.38, 18.75, 37.5, 75, 150, 300, 600, and 1200 µg/mL. The concentration of the solvents in the controls was also kept at 2× (5%) the final concentration. The fungicide solutions or solvents were diluted with potato dextrose broth (Difco^TM^ PDB; Becton, Dickinson and Company, Le Pont-de-Claix, France) (2× concentrated) in flat-bottomed 96-well plates (Fisher Scientific AG, Reinach, Switzerland) (total volume of 100 µL per well, with all concentrations in triplicate).

Overnight cultures of all the yeast isolates were prepared in PDB (3 mL, 22 °C, 200 rpm) using five yeast colonies maintained on PDA for 7 d after thawing from the 15% (*v*/*v*) glycerol stocks. The optical density at 600 nm (OD_600_) was measured using a spectrophotometer (GE Healthcare NovaspecTM III, Fisher Scientific AG, Basel, Switzerland), and yeast suspensions with final densities (OD_600_) of 1 were prepared. Of these yeast suspensions, 10 µL was added to each well. Each plate was closed with a lid and incubated in the dark for 72 h at 22 °C (shaking at 240 rpm). The OD_600_ at the 72 h time point was measured using a microplate reader (Spark^®^, Tecan Life Science AG, Männedorf, Switzerland) (set at 25 °C, 240 rpm (30 sec), 600 nm, and 10 flashes). These OD_600_ values were used to assess the minimum inhibitory concentrations (MIC_50_), which were defined as the lowest concentrations of the fungicides that resulted in a 50% reduction of yeast growth (as assessed by OD_600_ measurements). Each experiment was repeated at least three times.

### 4.4. Statistical Analyses

All the statistical analyses (unless otherwise specified) were performed in GraphPad Prism 8.4 (GraphPad software, San Diego, CA, USA), with the level of significance set to 0.05. The means and standard deviations of three technical replicates for each isolate were determined for each experiment (these were later used as the three experimental replicates). The MIC_50_ value was calculated by non-linear regression (curve fit) of the log concentrations against the normalised mean OD_600_ responses. Descriptive statistics (mean, median, and range) were calculated for the cleaned data (one outlier was removed based on Grubb’s test) [81] (pp. 26–28) of the calculated MIC_50_ values. The frequency distribution of the sensitivity for each fungicide was determined using the log-transformed MIC_50_ values, and the presence of a Gaussian normal distribution was tested according to the Shapiro–Wilk test.

The mean MIC_50_ values for the 30 *A. pullulans* isolates were compared with the mean EC_50_ values for plant pathogenic fungi (either *B. cinerea* or *Penicillium* spp.) using *t*-tests. The mean EC_50_ values for CYP (0.008 µg/mL) and CPN (0.9 µg/mL) for the control *B. cinerea* were calculated based on published values for 6 wild strains of *B. cinerea* [37,38]. The mean EC_50_ value for *Penicillium* spp. for DFN (0.16 µg/mL) was based on published data for 97 wild *Penicillium* spp. strains [28].

Simple linear correlation coefficients (Pearson’s r) [81] (p.92) were calculated to determine the relationships between the sensitivities of (a) DFN and CYP, (b) DFN and CPN, and (c) CYP and CPN. The log-transformed MIC_50_ values, which assumed a normal distribution, were used for correlation analysis. The clustering of the MIC_50_ values of all the isolates for the three fungicides was evaluated based on the log-transformed data. A hierarchical dendrogram was constructed a using complete-linkage clustering method with the Euclidean distance metric as a measure of the intervals between clusters in Morpheus (https://software.broadinstitute.org/morpheus). Evaluation for potential cross-resistance (possible similar mechanisms of resistance) was performed using the log MIC_50_ values.

### 4.5. Phylogenic Analysis

The ITS sequences of all 30 *A. pullulans* isolates and additional, already published, strains were selected and aligned using MUSCLE, built into MEGA, version 10.1 [36]. All regions were used without gap deletion during the alignment. The phylogenetic analysis involved 44 nucleotide sequences (30 *A. pullulans* sequences, 25 published sequences of other species within the genus *Aureobasidium*, and *Kabatiella bupleuri* (CBS 131304) as the outgroup). A phylogenetic tree was constructed with the maximum-likelihood algorithm using the Tamura–Nei Model [35], and the internal branch support was assessed based on 500-bootstrapped dataset. A discrete Gamma distribution was used to model evolutionary rate differences among sites, which showed some sites to be evolutionarily invariable ([+/], 0.00% sites). All positions with missing data were eliminated, while those with gaps were included, and in the final dataset, there were a total of 675 aligned positions (including gaps).

## Figures and Tables

**Table 1:** Yeast species isolated from soil, flower, leaf, and fruit samples in this study. *A. pullulans, Holtermaniella,* and *S. metaroseus* were isolated from all sample types. The species were identified by MALDI-TOF mass spectrometry (MS) as a fast and economical alternative to DNA sequencing. Isolates that could not be identified by MALDI-TOF MS were determined based on the ITS sequence. Those identified by the ITS sequence were assigned species hypotheses (SH numbers) [32,33].

Soil	Flower	Leaf	Fruit
*Aureobasidium pullulans*	*Aureobasidium pullulans*	*Aureobasidium pullulans*	*Aureobasidium pullulans*
*Holtermaniella*	*Holtermaniella*	*Holtermaniella*	*Holtermaniella*
*Sporidiobulus metaroseus*	*Sporidiobulus metaroseus*	*Sporodiobolus metaroseus*	*Sporidiobolus metaroseus*
*Metschnikowia pulcherrima*	*Metschnikowia pulcherrima*	*Metschnikowia pulcherrima*	
*Cryptococcus laurentii*	*Cryptococcus laurentii*		
*Cyberlindnera misumaiensis*	*Cyberlindneramisumaiensis*		
*Hanseniaspora uvarum*	*Hanseniaspora uvarum*		
*Cystofilobasidium macerans*	*Cystofilobasidium macerans*		
*Bullera alba*		*Bullera alba*	
*Sporidiobolaceae pararoseus*		*Sporodiobolaceae pararoseus*	
*Schwanniomyces capriottii*		*Schwanniomyces capriottii*	
*Rhodotorula*		*Rhodotorula*	
*Apiotrichum porosum*			
*Barnettozyma california*			
*Candida californica*			
*Coniochaeta*			
*Cryptococcus*			
*Cyberlindnera saturnus*			
*Cystofilobasidiacea*			
*Cystofilobasidium capitatum*			
*Dipodascaceae*			
*Dipodascus geotrichum*			
*Hanseniospora*			
*Kregervanrija fluxuum*			
*Pichia mandshurica*			
*Pichia sporocuriosa*			
*Pichia terricola*			
*Pichiaceae*			
*Saccharomycopsis*			
*Saccharomycopsis schoenii*			
*Saccharomycopsis vini*			
*Saitozyma podzolica*			
*Schwanniomyces pseudopolymorphus*			
*Sporidiobolaceae*			
*Tremellomycetes*			
*Trichosporon*			
*Wickerhamomyces anomalus*			
*Zygosaccharomyces microellipsoides*			
			
		*Erythrobasidium hasegawianum*	
		*Filobasidium*	
		*Filobasidium floriforme*	
		*Filobasidium magnum*	
		*Rhodotorula graminis*	
			*Curvibasidium*

**Table 2:** Overall MIC_50_ mean, median, and range for captan (CPN), cyprodinil (CYP), and DFN for all *A. pullulans* isolates.

Isolate Name	MIC_50_ µg/mL			Isolating Fungicide
	Captan	Cyprodinil	Difenoconazole	
F2	5.1	2.8	0.5	None
Fr1	22.5	9	1	None
Fr2	40	7.4	0.6	None
SFr4.3	25.1	7.9	2.4	Slick (DFN)
LSK 2.11	19.7	49.3	1.8	Slick (DFN)
FLSK 5.1	18.8	7.5	1.3	Slick (DFN)
LSK 10.4	41	18.9	2.4	Slick (DFN)
LCH 10.2	20.9	34.1	7.1	Chorus (CYP)
LCH 5.9	51.5	11.9	1.3	Chorus (CYP)
ChF4.2	6.3	2.2	0.9	Chorus (CYP)
LCH 2.1	44.7	29.7	3.7	Chorus (CYP)
CaL1.1	19.1	20.6	1.1	Captan 80 WD (CPN)
CaFr2.1	19.4	3.8	1.2	Captan 80 WD (CPN)
LC 5.2	39.5	186	0.7	Captan 80 WD (CPN)
LC 1.9	38.4	59.6	5.2	Captan 80 WD (CPN)
LC 1.3	50.8	50.5	4.2	Captan 80 WD (CPN)
LF 3.10	20.6	3.4	4.8	Flint (Trifloxystrobin)
LF 5.11	23.4	14.1	2.2	Flint (Trifloxystrobin)
FFr4.3	19.9	3.2	3.1	Flint (Trifloxystrobin)
LF 5.16	28.7	42.8	1.4	Flint (Trifloxystrobin)
LF 5.10	54.4	41.9	10.1	Flint (Trifloxystrobin)
AF4.1b	17	8.9	0.4	Amphotericin B
AL4e	19.3	1.45 × 10^39^	2.2	Amphotericin B
AF4.1a	5.8	3.6	0.6	Amphotericin B
CnF4.2	5.3	2	0.6	Capsofungin
CnL4a	21.4	6	1.7	Capsofungin
CnFr4.4	33.1	9	0.9	Capsofungin
FL4.31	40.2	8.3	1	Fluconazole
FLF 4.3	5.7	4.7	1.4	Fluconazole
FLS4a	109.6	6.6	1.8	Fluconazole
Mean	28.9	22.6 *	2.2	
Median	21.9	8.9	1.4	
Range	5.1–109.5	2.0–186 *	0.4–10.1	

* For CYP, isolate AL4e was excluded from the mean analysis because it was not controlled at any CYP concentration used here. The fungicides and their active compounds used for isolation are indicated.

**Table 3:** The 30 isolates of *A. pullulans*, with sample sources and times of sampling, used for quantifying the MIC_50_ for CPN, CYP, and DFN All the isolates were identified based on the ITS sequence, which resulted in the SH number SH1515060.08FU. Isolates FLSK5.1, ChF4.2, and LF5.10 were identified by MALDI-TOF MS.

No	Isolate Name	Sample	Season Isolated
1	F2	Flower	Spring
2	Fr1	Fruit	Summer
3	Fr2	Fruit	Summer
4	AF4.1b	Flower	Summer
5	AL4e	Leaf	Summer
6	AF4.1a	Flower	Spring
7	LF 3.10	Leaf	Autumn
8	LF 5.11	Leaf	Autumn
9	FFr4.3	Fruit	Summer
10	CaL1.1	Leaf	Summer
11	CaFr2.1	Fruit	Summer
12	LC 5.2	Leaf	Autumn
13	CnF4.2	Flower	Spring
14	CnL4a	Leaf	Summer
15	CnFr4.4	Fruit	Summer
16	FL4.31	Leaf	Summer
17	FLF 4.3	Leaf	Spring
18	FLS4a	Leaf	Spring
19	LCH 10.2	Leaf	Autumn
20	LCH 5.9	Leaf	Autumn
21	ChF4.2	Flower	Spring
22	SFr4.3	Fruit	Summer
23	LSK 2.11	Leaf	Autumn
24	FLSK 5.1	Leaf	Winter
25	LC 1.9	Leaf	Autumn
26	LC 1.3	Leaf	Autumn
27	LCH 2.1	Leaf	Autumn
28	LSK 10.4	Leaf	Autumn
29	LF 5.16	Leaf	Autumn
30	LF 5.10	Leaf	Autumn

## Supplementary Materials

The following is available online at https://www.mdpi.com/2079-6382/9/9/602/s1, Table S1: Yeast species obtained in the course of this study.
